# Fast Structural Search in Phylogenetic Databases

**Published:** 2007-02-20

**Authors:** Jason T. L. Wang, Huiyuan Shan, Dennis Shasha, William H. Piel

**Affiliations:** 1 Department of Computer Science, New Jersey Institute of Technology, University Heights, Newark, NJ, USA; 2 Department of Computer Science, New Jersey Institute of Technology, University Heights, Newark, NJ, USA; 3 Courant Institute of Mathematical Sciences, New York University, New York, NY, USA; 4 Department of Biological Sciences, State University of New York at Buffalo, Buffalo, NY, USA

**Keywords:** Structural pattern matching, structural search and retrieval, tree search strategies, phylogenetic trees

## Abstract

As the size of phylogenetic databases grows, the need for efficiently searching these databases arises. Thanks to previous and ongoing research, searching by attribute value and by text has become commonplace in these databases. However, searching by topological or physical structure, especially for large databases and especially for approximate matches, is still an art. We propose structural search techniques that, given a query or pattern tree P and a database of phylogenies D, find trees in D that are sufficiently close to P. The “closeness” is a measure of the topological relationships in P that are found to be the same or similar in a tree D in D. We develop a filtering technique that accelerates searches and present algorithms for rooted and unrooted trees where the trees can be weighted or unweighted. Experimental results on comparing the similarity measure with existing tree metrics and on evaluating the efficiency of the search techniques demonstrate that the proposed approach is promising.

## Introduction

Scientists model phylogenetic relations using unordered labeled trees and develop methods for constructing these trees (Berry and Bryant 1999; Camin and Sokal 1965; Felsenstein 2003; Gusfield 1997; Kannan et al. 1990; Wang et al. 2000). Different theories concerning the phylogenetic relationship of the same set of species often result in different phylogenetic trees. Even the same phylogenetic theory may yield different trees for different orthologous genes. With the unprecedented number of phylogenetic trees constructed based on these various theories, the need to analyze the trees and manage phylogenetic databases is urgent and great (Piel et al. 2003). One important problem in this domain is to be able to compare the trees, thus possibly determining how much two hypotheses have in common (Bryant et al. 2000; Cole et al. 2000; DasGupta et al. 1998; Kannan et al. 1995; Thorley and Page 2000). The common portion of two trees may represent added support for the phylogenetic relationship of the corresponding species.

Our motivation for studying the tree matching problem comes from the development of tools for analyzing the phylogenetic data. One particular tool we are developing is a system for searching phylogenetic trees. Given a query or pattern tree P and a set of data trees D, this structural search engine is able to find near neighbors of P in D where the similarity scores between those neighboring trees and P are greater than or equal to a user-specified value σ. Central to our search engine is an algorithm for computing the similarity score from P to each data tree D in D.

Our data consists of the phylogenetic trees stored within the widely used phylogenetic information system TreeBASE (Piel et al. 2003; Sanderson et al. 1994), accessible at http://www.treebase.org. These trees model the evolution history among life forms. The sampled life forms, whose biological characteristics are used to infer their evolutionary history, usually appear as leaf nodes. Each internal node of one such tree represents an inferred ancestor organism of the organisms represented by its child nodes. There can be multiple levels of ancestors, with multiple organisms sharing the same ancestors.

In Wang et al. (2003) we introduced a similarity measure to compare phylogenetic trees that satisfy the following properties:

- Each tree is rooted and unordered, i.e., the order among siblings is unimportant, and no weight is assigned to any edge of the tree.
- Each leaf node has a label and that label appears only once in the tree, though it may appear in other trees.
- Each non-leaf node either has a label that appears nowhere else in the tree or has no label. An unlabeled internal node stands for an unnamed evolutionary unit.
- Each unlabeled internal node has at least two children.

These properties characterize many trees in Tree-BASE and those generated by modern tree reconstruction programs.

In this paper we extend the work in Wang *et al*. (2003) to compare unrooted phylogenetic trees as well as weighted trees, i.e., trees whose edges have weights. We first review the similarity measure and search algorithms introduced in Wang *et al*. (2003) for rooted trees. We then discuss their extensions for unrooted trees and weighted trees. Next we compare the proposed similarity measures with existing tree metrics. Finally we present performance results for near neighbor searching and conclude the paper.

## Methods

### Up and Down Operations

Unless otherwise stated, trees discussed here refer to rooted unordered trees satisfying the four properties described in the Introduction section. We consider two types of operations, *up* and *down*, between any two nodes in a tree. These operations are intended to capture the hierarchical structure in the tree. If *v* is a child node of *u*, we use *v* ↑ *u* to represent an up operation from *v* to *u*, and use *u* ↓ *v* to represent a down operation from *u* to *v*. Then, for any pair of nodes *m*, *n* in the tree *T*, one can count the number of up and down operations to move, say a token, from *m* to *n.*

**Figure 1:**
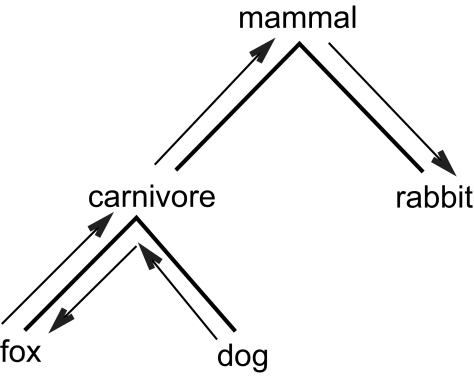
illustration of up and down operations between two nodes in a tree.

For example, consider the tree in Figure 1 and the two nodes “fox” and “rabbit” in the tree. It takes two up operations (“fox” ↑ “carnivore” and “carnivore” ↑ “mammal”) and one down operation (“mammal” ↓ “rabbit”) to go from “fox” to “rabbit” in the tree. As another example, it takes one up operation “dog” ↑ “carnivore”) and one down operation (“carnivore” ↓ “fox”) to go from “dog” to “fox” in the tree.

### Updown Matrix

Given a tree *T*, we can now build two matrices, referred to as the *Up matrix U* and the *Down matrix D*, of integer values where *U* [*u*, *v*] represents the number of up operations from node *u* to node *v* and *D* [*u*, *v*] represents the number of down operations from *u* to v in the shortest path connecting *u* and *v* in *T.* Obviously *U* [*u*, *u*] = *D* [*u*, *u*] = 0 for any node *u in T.*

**Figure 2:**
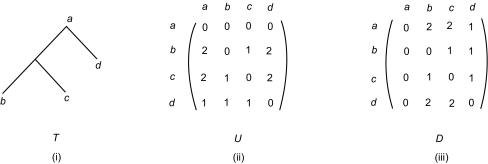
a tree and its up and down matrices.

Figure 2 shows a tree and its Up and Down matrices. Notice that one of the internal nodes, namely the parent of *b* and *c*, does not have a label. The unlabeled node does not appear in the matrices. It is easy to see that from matrix *U*, we can obtain matrix *D*, and vice versa. *D* is the transpose of *U* (or vice versa). We will therefore only use matrix *U* throughout the paper and refer to it as the Updown matrix. The Updown matrix of a tree *T* describes the structure of *T.* Computing the Updown matrix for a tree *T* requires **O**(*N*^2^) time where *N* is the number of nodes *in T.*

### Updown Distance

In general, when using a search engine, if the user inputs a query tree with three nodes “fox”, “dog” and “tiger” plus their parent node “mammal”, the user often expects to see data trees in search results containing these nodes. If the user doesn’t want to see a search result containing, for example, a node “tiger”, he or she can simply input a query tree having “fox”, “dog” and “mammal” only. This implies that in designing a search engine and a similarity or distance measure, the following two criteria should be considered together:

1. Whether all, or at least most of, the labeled nodes of the query tree *P* occur in a data tree *D*;
2. To which extent the query tree *P* is (dis)-similar to the data tree *D* in structure.

With these criteria in mind, we seek nodes in *D* that match nodes in *P* when comparing *P* with *D.* Specifically, let *V_P_* be the set of labeled nodes in *P* and let V*_D_* be the set of labeled nodes in *D.* Let *U_P_* represent the Updown matrix of *P* and let *U_D_* represent the Updown matrix of *D.* Let *I* denote the intersection of *V_P_* and *V_D_*; let *J* denote *V_D_*−*V_D_*.

We define the *Updown distance* from *P* to *D*, denoted *Updown dist* (*P*, *D*), as

(1) $$\begin{array}{l} Updown\_dist(P,D)=\Sigma_{\text{u}\in \text{I}}\Sigma_{\text{v}\in \text{I}}|U_{P}[u,v]-U_{D}[u,v]|\\ +\Sigma_{\text{u}\in \text{J}}\Sigma_{\text{v}\in \text{J}}U_{P}[u,v] \end{array}$$

The similarity score from P to D, denoted *USim* (*P*, *D*), is calculated by

(2) $$USim(P,D)=\left(1-\frac{Updown\_dist(P,D)}{\Sigma_{\text{u}\in \;{\text{v}}_{\text{P}}}\Sigma_{\text{v}\in {\text{v}}_{\text{P}}}U_{P}[u,v]}\right)\times 100\%$$

The similarity score from *P* to *D* is a measure of the topological relationships in *P* that are found to be the same or similar in *D*. If *P* and *D* are the same or if one can find a substructure in *D* that exactly matches *P*, then *USim* (*P*, *D*) = 100%. On the other hand, if *P* and *D* do not have any labeled node in common, then *USim* (*P*, *D*) = 0. The time complexity of the algorithm for computing *USim* (*P*, *D*) is **O**(*M*^2^ + *N*) where *M* is the number of nodes in *P*, and *N* is the number of nodes in *D.*

## Tree Reduction

**Figure 3:**
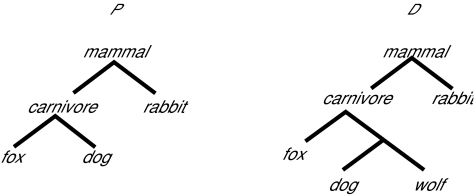
example trees.

Figure 3 shows a query tree *P* and a data tree *D* that satisfy the four properties described in the Introduction section. In the biological sense, when comparing *P* with *D*, the similarity score *USim* (*P*, *D*) should be 100%. Motivated by this example, we incorporate a *data tree reduction* technique into our structural searching algorithm, which works as follows.

Consider a query tree *P* and a data tree *D* and their Updown matrices. Find the column and row indexes of the nodes in the intersection of *V_P_* and *V_D_*. Mark those matching nodes in *D* with asterisks. If two distinct nodes of *D* are marked, then their least common ancestor is also marked. We then consider the reduced data tree *D*′ *of D* that contains only the marked nodes. Equivalently, we remove unmarked nodes having only one neighbor (this must preserve connectedness). The above removal might yield additional unmarked nodes with one neighbor, which themselves will be removed. If an unmarked node *n* is connected to two other nodes *m*_1_ and m_2_, then remove *n* and link *m*_1_ and *m*_2_. This too preserves connectedness. Continue doing these two operations until neither can be done. The node removal operation is similar to the “degree-2 delete” operation defined in Wang *et al*. (2002) where a node can be deleted when the node’s degree is less than or equal to 2. Notice that after reduction, the Updown matrices will change, and we use the new matrices to calculate the Updown distance and similarity score from *P* to *D.*

**Figure 4:**
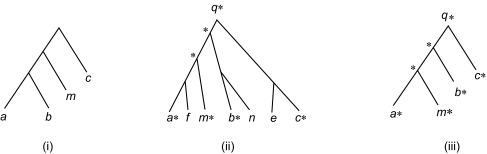
example showing how the data tree reduction technique works in near neighbour searching.

Figure 4 presents an example. In the figure, (i) shows a query tree, (ii) shows a data tree in which some nodes are marked, and (iii) shows the reduced tree of the data tree in (ii). In performing a structural search, our algorithm first applies the tree reduction technique to a data tree *D*, and then calculates the similarity score from the given query tree *P* to the reduced tree of *D* using the formula in Equation (2). The resulting value is then presented as the similarity score from *P* to *D.*

For example, in Figure 4, the similarity score from the query tree in (i) to the reduced data tree in (iii) is 68.42%. Hence, our algorithm displays the data tree in (ii) and indicates that the similarity score from the query tree to the data tree is 68.42%. This matching technique yields a similar effect as tree matching with variable length don’t cares (Page 2005b; Shasha *et al*. 2002), though the proposed approach does not require the user to explicitly specify the don’t cares in the query tree.

### A Filter

Given a query or pattern tree *P* and a database of phylogenies *D*, our goal is to find near neighbors of *P* in *D* where the similarity scores between the near neighbors and P are greater than or equal to a user-specified threshold σ. We develop a filter to speed up the search, which works as follows. For the database of trees, we create a hash table keyed by pair of node labels and each hash bin contains tree identification numbers. The pair can be in alphabetical order because *U* [*u*, *v*] = *D* [*v*, *u*] for any pair of node labels (*u*, *v*). Now given the query tree *P*, we consider each pair of node labels in P and see which trees of the database the pair is in. (This requires time independent of the size of the database.) Sort the data trees by the number of hits.

When evaluating a data tree *D*, we get a lower bound on the Updown distance from *P* to *D* by looking at *U_P_* [*u*, *v*] where *U_P_* is the Updown matrix of *P* and (*u*, *v*) is a pair in *P* that is missing from *D.* The lower bound, denoted *Low*, is computed by summing up *U_P_*[*u*, *v*] for all pairs of (*u*, *v*) *of P* that are missing from *D.* From the lower bound, we can calculate an upper bound, denoted *U_PP_*, on the similarity score from *P* to *D*, where and *V_P_* is the set of labelled nodes *in P.*

(3) $$Upp=\left(1-\frac{Low}{\sum_{\text{u}\in \;{\text{V}}_{\text{P}}}\sum_{\text{v}\in {\text{V}}_{\text{P}}}U_{P}[u,v]}\right)\times 100\%$$

If the upper bound is already smaller than the user-specified value σ, we can eliminate *D* from consideration without calculating the similarity score from *P* to *D.* Furthermore, if a data tree *D* has a set *S* of *k* hits and it is decided *D* doesn’t qualify to be a solution after calculating the similarity score from *P* to *D*, then any data tree *D*′ that only has S′ of *k*′ hits, where *k*′ < *k* and *S*′ is a subset of *S*, will not be a solution and hence can be eliminated from consideration. As our experimental results show later, this filtering technique works well in practice.

### Extensions to Weighted and Unrooted Trees

Some tree reconstruction methods provide information to build a weighted tree where the weight on an edge represents the estimated evolutionary distance between the two nodes connected by the edge (Page and Holmes 1998). In extending our approach for weighted trees, we associate each up and down operation with a weight that equals the weight of the corresponding edge. Instead of having *U* [*u*, *v*] represent the number of up operations from node *u* to node *v*, we use *U* [*u*, *v*] to represent the sum of weights associated with the up operations from *u* to *v*. Likewise, we use *D* [*u*, *v*] to represent the sum of weights associated with the down operations from *u* to *v*. The similarity score between two weighted trees is then calculated in the same way as in Equation (2).

Some phylogenetic tree reconstruction methods may produce unrooted unordered trees, or free trees. An unrooted tree is one that specifies only kinship relationships among taxa without specifying ancestry relationships. The common ancestor of all taxa is unknown. Each edge in an unrooted tree can be weighted or unweighted. Let *T be* an unrooted unordered tree. We define the *Additive matrix A* for *T* where each entry *A* [*u*, *v*] is the sum of the edge weights on the shortest path connecting *u* and *v* in *T.* If *T* is not weighted, then *A* [*u*, *v*] is simply the number of edges on the shortest path connecting *u* and *v* in *T* (reminiscent of the additive distance for an unrooted tree described in Berry and Bryant 1999; Buneman 1971; Wang and Gusfield 1998). Notice that when a rooted tree is treated as unrooted, we have *U* [*u*, *v*] + *U* [*v*, *u*] = *A*[*u*, *v*] for all pairs of (*u*, *v*) in the tree, where *U* and *A* are the Updown and Additive matrices respectively. Therefore, matrix *A* can be obtained from *U* (the converse is not true). As matrix *A* is an additive matrix, the four-point condition (Buneman 1971; Zaretskii 1965) applies. Hence, an Updown matrix corresponds to a unique Additive matrix which corresponds to a unique tree. This holds for both weighted and unweighted trees.

Now let *A_P_* represent the Additive matrix of the query tree *P* and let *A_D_* represent the Additive matrix of a data tree *D.* Let *V_P_* *be* the set of labelled nodes in *P* and let *V_D_* *be* the set of labelled nodes in *D.* Let *I* be the intersection of *V_P_* *and V_D_*; let *J* denote *V_P_*−*V_D_*. We define the Additive distance from *P* to *D*, denoted *Add_dist* (*P*, *D*), as follows (reminiscent of the measure defined in Williams and Clifford 1971):

(4) $$\begin{array}{l} Add\_dist(P,D)=\Sigma_{\text{u}\in \text{I}}\Sigma_{\text{v}\in I}|A_{P}[u,v]-A_{D}[u,v]|\\ +\Sigma_{\text{u}\in \text{J}}\Sigma_{\text{v}\in J}[u,v] \end{array}$$

The similarity score from *P* to *D*, denoted *ASim* (*P*, *D*), is calculated by

(5) $$ASim(P,D)=\left(1-\frac{Add\_dist(P,D)}{\Sigma_{\text{u}\in \;{\text{v}}_{P}}\Sigma_{\text{v}\in {\text{v}}_{P}}A_{P}[u,v]}\right)\times 100\%$$

The time complexity of the algorithm for computing *ASim* (*P*, *D*) is **O**(*M*^2^ + *N*) where *M* is the number of nodes in *P*, and *N* is the number of nodes in *D.* It can be shown that for two unrooted trees *P* and *D*, whether they are weighted or unweighted, *P* and *D* are identical if and only if the similarity score from *P* to *D* is 100%. This property holds for rooted trees as well.

## Experiments and Results

### Comparison of (Dis)similarity Measures

To evaluate the quality of the proposed similarity measures, we compared *USim* defined in Equation (2) with four widely used tree metrics implemented in the COMPONENT tool (Page 2005a). These tree metrics include partition metric (PAR), nearest neighbour interchange metric (NNI), quartet metric (QUA) and maximum agreement subtree metric (MAST). Specifically, we compared the distribution of the metric values on 945 unweighted rooted trees generated by the COMPONENT tool. The query tree was generated randomly; the 945 data trees covered the entire tree space of unweighted rooted trees with 6 labels. We compared the query tree with each data tree to obtain a metric or (dis)similarity value. For PAR, the metric value equals the number of edges in the query tree for which there is no equivalent (in the sense of creating the same partitions) edge in the data tree. For NNI, the metric value equals the number of nearest neighbour interchange operations needed to transform the query tree to the data tree. For QUA, the metric value equals the proportion of quartets that are shared in the query tree and the data tree. For MAST, the metric value equals the number of leaves removed to obtain a maximum agreement subtree of the query tree and the data tree.

**Figure 5:**
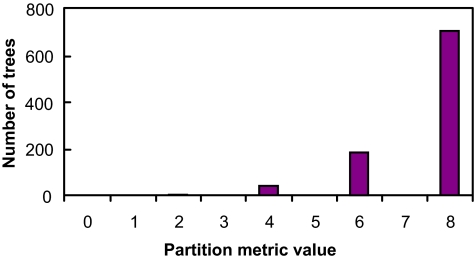
distribution of PAR metric values.

**Figure 6:**
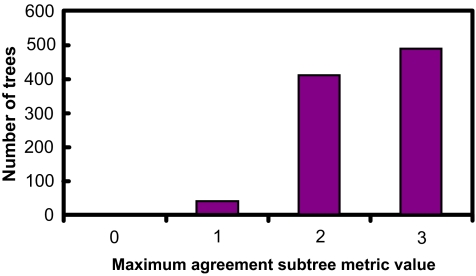
distribution of MAST metric values.

**Figure 7:**
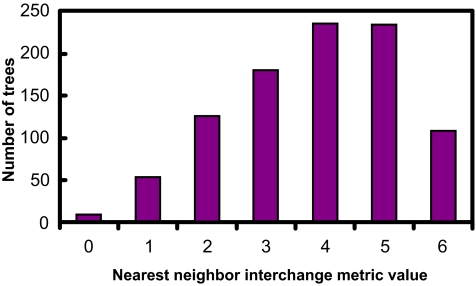
distribution of NNI metric values.

**Figure 8:**
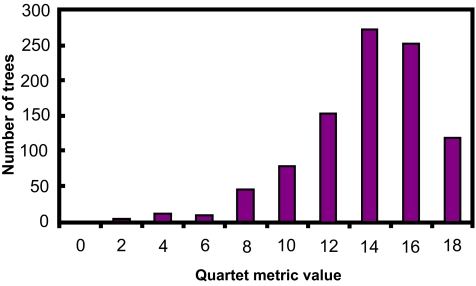
distribution of QUA metric values.

**Figure 9:**
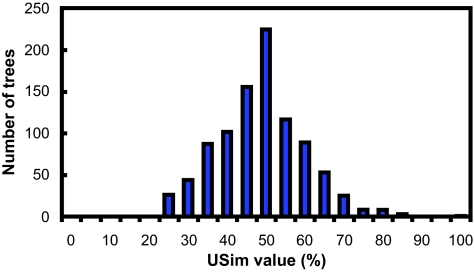
distribution of *USim* values.

Figures 5–9 summarize the experimental results. In each figure, the X-axis shows different metric values. For each specified value on the X-axis, the figure shows the number of data trees whose metric/(dis)similarity value from the query tree equals the specified value. The pattern in Figures 5 and 8 agrees with the finding reported in Steel and Penny (1993), which presented a similar simulation. We see from Figures 5–9 that the proposed similarity measure has a good distribution of values, unlike partition metric (PAR) and maximum agreement subtree metric (MAST). It should be pointed out that each tree metric has its own advantages and shortcomings. As far as structural search is concerned, it is desirable to have a tree metric with a wide range of values. This would produce a sensible, ranked list of search results. We have also tested additional query trees. The distributions of metric values depend on the tested query trees, though the qualitative conclusion obtained from these additional experiments remains the same.

Table 1 shows an in-depth comparison between the four widely used tree metrics and the proposed similarity measures *USim* and *ASim*, collectively referred to as WSSP. In the table, a “Y” value in the “Polynomial computable” column means that there is a polynomial time algorithm for computing the corresponding tree metric and an “N” value means that computing the corresponding tree metric has been shown to be NP-hard. From Table 1 it can be seen that the running time of WSSP is better than NNI (nearest neighbour interchange metric). WSSP can be applied to weighted trees and unweighted trees where trees can be fully resolved or unresolved. It can be used to compare two trees whose internal nodes have labels and whose leaves have different taxa as shown in Table 1. The bottom line is that WSSP could be a useful metric in addition to the other excellent ones available.

### Efficiency of the Filter and Search Method

**Figure 10:**
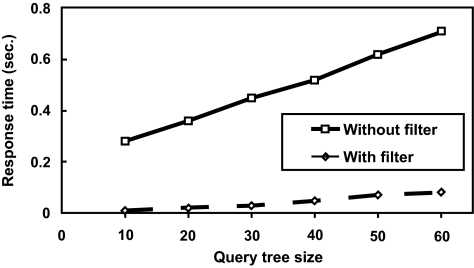
running times on 1,000 synthetic trees for search methods with and without the filter.

**Figure 11:**
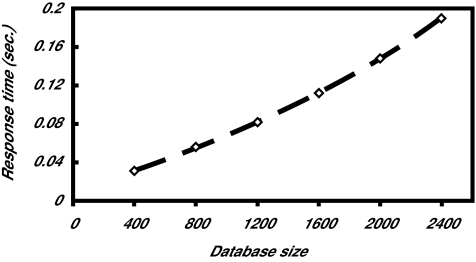
running times of the proposed search method on different sizes of databases.

We have also tested our filter technique on synthetic data. One thousand unweighted rooted trees were randomly generated, each tree having 100 nodes. The string labels of nodes were randomly chosen from a dictionary of size 500. The threshold value σ was set to 60%. In each run, a tree was selected and modified into the query tree and the other trees were used as data trees. 1,000 runs were tested and the average was plotted. Figure 10 shows the results for varying query tree sizes. It can be seen from the figure that the proposed filter speeds up searches considerably. It was also observed that the running time drops as the user-specified threshold value σ increases. This happens because fewer data trees survive the filter when σ becomes larger. Figure 11 shows that the proposed search method scales up well – its running time increases linearly with increasing number of trees. These results are consistent with those for real phylogenetic trees.

### A Structural Search Engine

**Figure 12:**
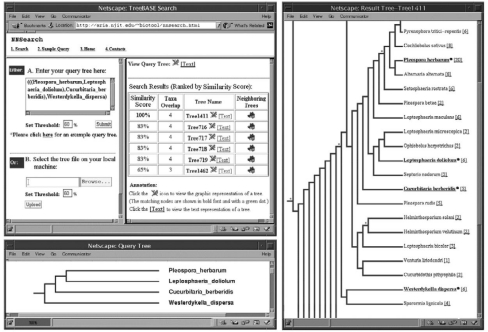
an example query and search results displayed via the Web-based interface of the proposed search engine.

The proposed search method for unweighted rooted trees has been implemented into a Web-based system connected with TreeBASE. Figure 12 shows the system’s main screen and query interface (the upper left window), a query tree (the lower left window), and the query tree’s nearest neighbor in TreeBASE (the right window). In the main screen, the query tree is expressed in the parenthesized string notation; in the other two windows this same query tree and the nearest neighboring tree are viewed in the dendrogram format.

Figure 12 displays data trees in TreeBASE where the similarity score, *USim*, of each data tree to the query tree is greater than or equal to the user-specified threshold, 60%. Among the data trees, Tree1411 is ranked highest, which is the nearest neighbor of the query tree with a 100% similarity score. It should be pointed out that after applying the tree reduction technique to Tree1411, the reduced tree is exactly the same as the query tree. (The matched taxa between the query tree and Tree 1411 are highlighted with a bullet and underscored in the figure.) Consequently the similarity score for Tree1411 is 100%.

This structural search engine is implemented using Java, HTML, Perl, CGI, and C. It is fully operational and is accessible at http://aria.njit.edu/~biotool/nnsearch.html. As of June 2005, about 600 users worldwide have accessed the search engine over 8,000 times totally. Most submitted query trees are small trees with 20 or fewer nodes. With these query trees, a moderate similarity score (e.g. 60%), and the approximately 1,600 unweighted rooted trees in TreeBASE, the system can perform a search in about one second on a SUN Ultra 20 workstation.

## Discussion

Unlike many existing metrics (Brodal *et al*. 2001; Brown and Day 1984; Cole *et al*. 2000; Day 1985; Hein *et al*. 1996; Kao *et al*. 1997; Kubicka *et al*. 1995; Lam *et al*. 1996; Page 1989; Page and Charleston 1998), designed for comparing two trees possibly with some constraints (e.g. the two trees must have the same set of leaves), the similarity scores described in the paper are mainly developed for near neighbor searching in phylogenetic databases. The similarity scores are not symmetric, i.e. *USim*(*X*, *Y*) ≠ *USim*(*Y*, *X*), *ASim*(*X*, *Y*) ≠ *ASim*(*Y*, *X*), for any two trees *X* and *Y.* The non-symmetry property is good in query-driven phylogenetic information retrieval; it distinguishes between the situation in which *X* is a query and *Y* is a data tree and the situation in which *Y* is a query and *X* is a data tree.

It should be pointed out that when a substructure in a data tree *D* exactly matches a query tree *P*, *USim*(*P*, *D*) = 100%, but the converse is not true. For example, if *P* = ((a, *b*), (*c*, *d*)) and *D* = ((a, *b*), *c*), the similarity score will be smaller than 100% despite the fact that a substructure of *P* exactly matches *D.* On the other hand, if *D* = ((*a*, *b*), (*c*, *d*)) and *P* = ((a, *b*), *c*), then the similarity score yields 100%. Moreover, the similarity score from *P* to *D* strongly depends on the size of the subset of taxa that are in the query tree but not in the data tree—the larger this subset, the smaller the similarity.

We have analyzed about 1,000 typical query trees submitted to our search engine by users around the world. Most query trees are small trees with 20 or fewer nodes and they may not have the same taxa as the data trees in TreeBASE. The users expect to see that a top ranked data tree in search results should be close to a query tree both in structure and in the number of overlapping taxa. Based on the user feedback, we designed the proposed similarity measure and ranking algorithm. On the other hand, if the user is only interested in evolutionary relations between species (i.e., tree topologies), the overlap between the taxa set of a query tree and that of a data tree is less important. In situations where the query tree and the data tree have the same set of taxa, the lower and upper bound that define the proposed filter would be 0 and 100% respectively, yielding a less efficient filter method.

In summary, we have presented a new approach to near neighbor searching for phylogenetic trees. Given a query or pattern tree *P* and a database of trees *D*, the proposed approach finds data trees *D* in *D* where the similarity score *of P* to *D* is greater than or equal to a user-specified threshold value. We developed similarity measures for comparing rooted and unrooted trees where the trees can be weighted or unweighted. The proposed algorithms have been used for analyzing the structures of phylogenetic trees and for performing structure-based searches in TreeBASE.

## Figures and Tables

**Table 1:** comparison of the five studied tree metrics

Metric	Weighted trees	Internal lables	Unresolved trees	Different taxa		Polynomial computable
PAR	N	N	Y	N	Y	(Page 2005a)
MAST	N	Y	N	Y	Y	(Steel and Warnow 1993)
NNI	N	N	N	N	N	(DasGupta et al 1995)
QUA	N	N	Y	N	Y	(Bryant et al 2000)
WSSP	Y	Y	Y	Y	Y	
